# Molecular detection of intestinal helminths and protozoa among young children in Dosso Region, Niger

**DOI:** 10.12688/gatesopenres.13124.2

**Published:** 2020-08-20

**Authors:** Kristen Aiemjoy, Ahmed M. Arzika, Catherine Cook, Elodie Lebas, Nils Pilotte, Jessica R. Grant, Steven A. Williams, Thomas M. Lietman, Jeremy D. Keenan

**Affiliations:** 1 Department of Medicine, Division of Infectious Diseases and Geographic Medicine, Stanford University School of Medicine, Stanford, California, USA; 2 The Carter Center, Niamey, Niger; 3 Francis I. Proctor Foundation for Research in Ophthalmology, University of California San Francisco, San Francisco, California, USA; 4 Department of Biological Sciences, Smith College, Northampton, Massachusetts, USA; 5 Department of Molecular and Cellular Biology, University of Massachusetts, Amherst, Massachusetts, USA

**Keywords:** Schistosoma, soil-transmitted helminths, protozoa, molecular diagnostics

## Abstract

Eukaryotic parasites are significant contributors to childhood illness in Niger. While helminthiases have received national attention through mass deworming efforts, the epidemiology of intestinal protozoa in Niger remains underexamined. This study employed real-time PCR diagnostics to describe the prevalence of two schistosomes, four soil-transmitted helminths, and one protozoan parasite in Boboye Department, Dosso Region. Prevalence was assessed using bulk stool specimens collected from a population-based sample of 86 children residing in 9 communities. Anthropometric measurements were used to calculate child growth *z*-scores and stool consistency was graded. Helminths were absent from the study population, with the exception of a single *Schistosoma haematobium* infection (1/86; 1.2%). *Giardia duodenalis* was the only protozoa present, detected in 65% (56/86) of children. Prevalence of *G. duodenalis* peaked in 2-year-olds with 88% (15/17) positivity. The population was generally undernourished, though growth indices did not differ significantly between children with and without *G. duodenalis* infection.

## Introduction

Eukaryotic parasites are significant contributors to childhood illness in Niger. In 2012, the World Health Organization estimated that 10–49% of Nigeriens lived with intestinal or urogenital schistosomiasis, while more than two-thirds of children required preventative chemotherapy for soil-transmitted helminths (STH) ^1^. In 2015, over 75% of pre-school aged children across Niger were targeted for preventative anthelmintic treatment ^2^. While mass deworming programs have drawn attention to the public health significance of helminthiases, the epidemiology of intestinal protozoa in Niger is not well described ^3^. However, limited data suggest that protozoa may cause an appreciable fraction of clinically evaluated enteric infections ^4, 5^.

Real-time polymerase chain reaction (qPCR) assays targeting high-copy-number genetic elements have been validated for many globally burdensome parasites and have been shown to provide greater sensitivity and specificity than traditional copromicroscopy ^6– 8^. This study makes use of these significant strides in diagnostic accuracy to evaluate the age-dependent prevalence of seven eukaryotic pathogens in nine rural communities of the Niger River Valley.

## Methods

### Study background

We conducted a cross-sectional study to evaluate the prevalence of helminthiasis, schistosomiasis and intestinal protozoa infection in Boboye Department, Dosso Region, Niger. The study was nested within the *Macrolides Oraux pour Réduire les Décès avec un Oeil sur la Résistance* (MORDOR) trial, a cluster-randomized trial investigating the effects of community mass administration of azithromycin on child health and mortality (Clinicaltrials.gov ID NCT02048007). Details of the MORDOR study design are available elsewhere ^9^. 

**Figure 1.**
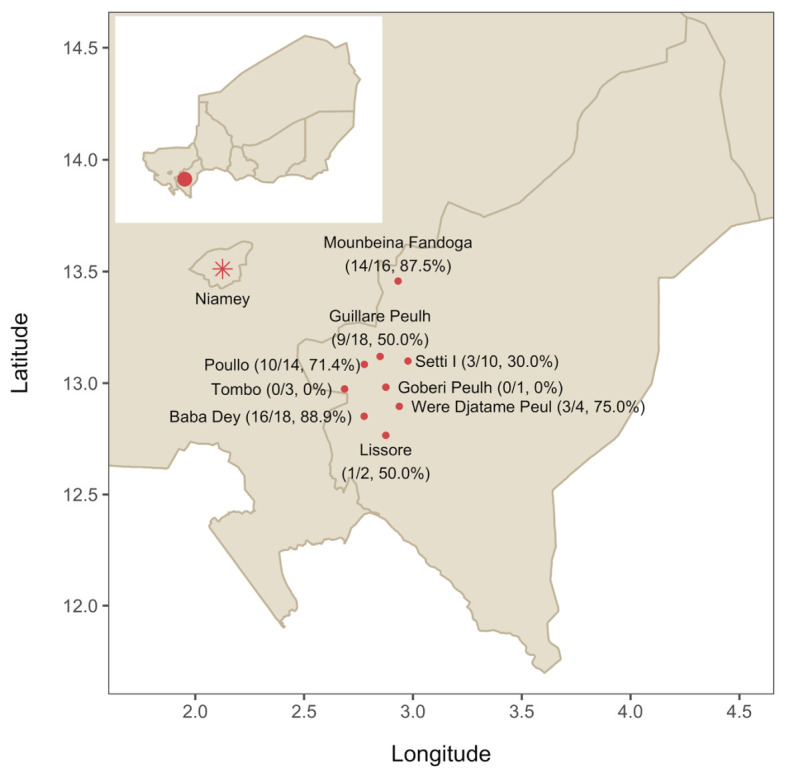
*Giardia duodenalis* prevalence by study site in Dosso Region, Niger. Study communities are indicated by red dots on the map. The prevalence is listed as the number of samples that tested positive for *Giardia duodenalis* over the total number of samples tested followed by the percentage.

This nested sub-study occurred in 9 of the 30 MORDOR study communities (see Figure 1) in Niger during a regularly scheduled study visit between May and June of 2017. These 9 communities were included because at the time this nested sub-study commenced there were only 9 study communities remaining for the annual site visit. All children residing in the study communities who were sampled for participation in the parent trial were eligible to participate. In the 9 communities, 447 children age 0–4 years were eligible to participate and 354 participated in the MORDOR study visit and 86 were able to provide a same-day bulk stool sample.

### Sample collection and analysis

On the day of the study visit, participating children gathered in a central location in the community. Trained field examiners performed three height and weight measurements per child in accordance with standard World Health Organization (WHO) protocols ^10^. Stool was collected at the time of the study visit, by instructing caregivers to have their children defecate in a potty chair lined with a plastic bag. After defecation, the caregiver returned the stool sample to the field collection team. The field examiner then collected a 0.5-mL specimen and placed it in an empty sterile 2-mL tube. No media was added to the samples. The stool samples were immediately placed on ice and transported to a -20°C freezer by the end of the day.

Isolation of total DNA from bulk stool and rectal swabs followed a procedure optimized for the qPCR detection of intestinal helminths ^11^. Multi-parallel qPCR ^7^ targeted the following species: the STH *Ancylostoma duodenale* ^6^ *, Ascaris lumbricoides* ^12^ *, Necator americanus* ^6^, and *Trichuris trichiura* ^6^; the trematode flukes *Schistosoma haematobium* ^13^ and *Schistosoma mansoni* ^13^; and the protozoan parasite *Giardia duodenalis* ^14^. All qPCR assays targeted highly repetitive non-coding elements with the exception of the *G. duodenalis* assay, which targets the small subunit ribosomal RNA gene. Procedures for sample collection, DNA isolation, qPCR, and quality control followed previously described protocols and standards ^11^.

The median height and weight measurements were used to calculate height-for-age (HAZ), weight-for-age (WAZ), and weight-for-height (WHZ) *z*-scores according to WHO child growth standards ^15^. We used generalized estimating equations to evaluate differences in anthropometry z-scores according to *G. duodenalis* positivity adjusting for age and accounting for clustering by community. All analyses were run in R v3.5.3.

### Ethical approval

Ethical committees from the Niger Ministry of Health and the University of California (San Francisco, CA, USA) granted approval for this study. Verbal informed consent was obtained in French from all caregivers. Verbal consent was obtained rather than written consent because of mixed literacy levels in the study population.

## Results

Stool samples were collected from 86 children residing in 9 communities. The median age was 2 years old (IQR 1-4). Overall, 59.3% (51/86) of participants were female. The mean child growth *z*-scores were less than zero for all indicators (HAZ = -1.53 [SD 1.39], WAZ = -1.55 [SD 1.22], WHZ = -0.97 [SD 1.1]).

*G. duodenalis* infection was detected in 65% (56/86) children living in 7 of the 9 surveyed communities. The 2 communities for which no infections were observed, Goberi Peulh and Tombo, only contained 1 and 3 total participants, respectively. For the 7 communities in which *G. duodenalis* was detected, prevalence ranged from 30% (3/10) to 89% (16/18) ( Figure 1). The prevalence of *G. duodenalis* increased with age, with 88.2% (15/17) of two-year-old children testing positive. The median Cq value for *G. duodenalis* positive stool was 29 (IQR 25.2, 31.7). Only 1 helminth infection was detected ( *S. haematobium*) ( Table 1).

**Table 1.** Prevalence of intestinal parasites among young children in Boboye Department, Dosso Region, Niger.

Variable	Age of child					
	< 1 year	1 year	2 years	3 years	4 years	Total
*N* children	11	20	17	13	25	86
Helminths						
* Ancylostoma duodenale*	0 (0%)	0 (0%)	0 (0%)	0 (0%)	0 (0%)	0 (0%)
* Ascaris lumbricoides*	0 (0%)	0 (0%)	0 (0%)	0 (0%)	0 (0%)	0 (0%)
* Necator americanus*	0 (0%)	0 (0%)	0 (0%)	0 (0%)	0 (0%)	0 (0%)
* Schistosoma haematobium*	0 (0%)	0 (0%)	0 (0%)	0 (0%)	1 (4%)	1 (1.2%)
* Schistosoma mansoni*	0 (0%)	0 (0%)	0 (0%)	0 (0%)	0 (0%)	0 (0%)
* Trichuris trichiura*	0 (0%)	0 (0%)	0 (0%)	0 (0%)	0 (0%)	0 (0%)
Protozoa						
* Giardia duodenalis*	6 (54.5%)	9 (50.0%)	15 (88.2%)	10 (76.9%)	16 (76.0%)	56 (65.1 %)

Children who tested positive for *G. duodenalis* had a -0.18 (95% CI: -0.77–0.40) lower height-for-age Z-score, a 0.22 (95% CI: -0.3–0.75) higher weight-for-age z-score and a 0.52 (-0.07–1.1) higher weight-for-height Z-score. However, none of these differences were statistically significant at the 0.05 level. Individual-level de-identified anthropometric, demographic and infection data are available (see *Underlying data*) ^16^.

## Discussion

We found that young children residing in the MORDOR study area in rural Niger had a high prevalence of *G. duodenalis* and a low prevalence of helminthic infections as measured by PCR in stool specimens. The population was generally undernourished, with all three anthropometric indices below average. Lower HAZ scores were observed among children positive for *G. duodenalis*, similar to prior observations in rural Amhara Region, Ethiopia ^17^. However, there was no significant difference in growth indices between children with and without *G. duodenalis* infection, though this could be related to sample size.

The administrative Region of Dosso is situated in the Niger River Valley, a more densely populated region with higher rates of infectious disease than the majority of the nation ^18^. In 2016, children 4 years of age and younger represented 81.1% of diarrhea cases, 43.7% of dysentery cases, and 37.7% of intestinal parasitisms recorded in Dosso Region, yet the etiological agents of these complaints remain unreported ^4^. In other rural regions of sub-Saharan Africa the prevalence of giardia in young children has been reported to be as high as 56% *when using a molecular assay on stool* ^3^. In a 2016 *cross-sectional sero-survey* in the Amhara region of Ethiopia over 80% of two year old children were seropositive for *G. intestinalis* ^19^. Risk factors for giardia infection include close contact with animals and animal feces and manure, residing in a rural area, drinking contaminated water and eating raw fruit ^3^. Given the high prevalence of *G. duodenalis* observed in the present study – albeit within a limited sample –the contributions of *G. duodenalis* infection to public health in the Niger River Valley warrant further investigation.

The low prevalence of STH and *S. mansoni* and *S. haematobium* in this cohort may relate to the success of national mass drug administration programs. In 2015, over 800,000 citizens of Dosso Region received praziquantel through mass drug administration, including over 200,000 living in Boboye Department; over half a million received albendazole, with over 100,000 residing in Boboye Department ^4^. However, without data on baseline prevalence and community-level anthelmintic distribution in this population, conclusions cannot be drawn. Another possible explanation for the low prevalence of *S. haematobium* may be that we tested stool rather than urine, the standard specimen type for this species. Though *S. haematobium* predominantly evacuates via urine, this species was detected in the feces of a single child. Ectopic elimination in the brain and intestine has been observed in *S. haematobium*, sometimes attributed to high parasite loads ^20^. Whether a greater number of children would have tested positive for *S. haematobium* by urine analysis cannot be known but could be an area of further research.

These findings indicate that *G. duodenalis* infections may be a significant contributor to child morbidity in the Dosso Region of Niger.

## Data availability

### Underlying data

Open Science Framework: Molecular detection of intestinal helminths and protozoa among young children in Dosso Region, Niger. https://doi.org/10.17605/OSF.IO/FMTYH ^16^.

This project contains the following underlying data:

NigerParasitePCR.csv. (Demographic, anthropometric variables and infection status for each participant.)

Data are available under the terms of the Creative Commons Attribution 4.0 International license (CC-BY 4.0).
