# Supplementary material for: Molecular detection of intestinal helminths and protozoa among young children in Dosso Region, Niger
Source: Gates Open Res. 2020 Aug 20;4:38. Originally published 2020 Apr 23. [Version 2] doi: 10.12688/gatesopenres.13124.2 (PMC7468184; doi:10.12688/gatesopenres.13124.2)
Supplement: Peer review history

# Peer review: Reviewer response for version 2

**DOI:** 10.21956/gatesopenres.14376.r29413

**Published:** 2020-09-02

**Authors:** Oumar Gaye

**Affiliations:** 1 University Cheikh Anta Diop de Dakar, Dakar, Senegal

We have evaluated the revision version of the article submitted and can confirm that the corrections are appropriate.

Is the work clearly and accurately presented and does it cite the current literature?

Yes

If applicable, is the statistical analysis and its interpretation appropriate?

Yes

Are all the source data underlying the results available to ensure full reproducibility?

Yes

Is the study design appropriate and is the work technically sound?

Partly

Are the conclusions drawn adequately supported by the results?

Yes

Are sufficient details of methods and analysis provided to allow replication by others?

Yes

Reviewer Expertise:

NA

I confirm that I have read this submission and believe that I have an appropriate level of expertise to confirm that it is of an acceptable scientific standard.

# Peer review: Reviewer response for version 2

**DOI:** 10.21956/gatesopenres.14376.r29414

**Published:** 2020-08-21

**Authors:** Olusola Ojurongbe

**Affiliations:** 1 Department of Medical Microbiology and Parasitology, Ladoke Akintola University of Technology, Osogbo, Nigeria

No further comments.

Is the work clearly and accurately presented and does it cite the current literature?

Yes

If applicable, is the statistical analysis and its interpretation appropriate?

Yes

Are all the source data underlying the results available to ensure full reproducibility?

Yes

Is the study design appropriate and is the work technically sound?

Partly

Are the conclusions drawn adequately supported by the results?

No

Are sufficient details of methods and analysis provided to allow replication by others?

No

Reviewer Expertise:

Molecular Parasitology and genetics.

I confirm that I have read this submission and believe that I have an appropriate level of expertise to confirm that it is of an acceptable scientific standard.

# Peer review: Reviewer response for version 1

**DOI:** 10.21956/gatesopenres.14306.r29090

**Published:** 2020-07-29

**Authors:** Oumar Gaye, Doudou Sow

**Affiliations:** 1 University Cheikh Anta Diop de Dakar, Dakar, Senegal; 2 University Gaston Berger of Saint-Louis, Saint-Louis, Senegal

**Regarding the question 2 on methodology and results interpretation, authors should take into account comments below:**

**Methods section**

1. How did authors select the 9 study communities among the 30 MORDOR communities? To be clarified.
2. Author must explain how did they choose the 86 children enrolled in this study among the 354 MORDOR participants, or it was an arbitrary choice? To be clarified.
3. Why did authors targeted *S. haematobium* in stool samples? *S haematobium* is responsible of urogenital schistosomiasis and can be found in some cases in stool samples. Did they have previous data showing the importance of this parasite in stool samples?
4. In the reference 14, the authors mention positive controls but it is not clear what the positive controls were.
5. It is not considered appropriate to consider Ct values above 38 as negative, high Ct values can be an indicator of an (upcoming) problem of PCR contamination or non-specificity of the PCR. 

**Results section**

1. Authors did not show any results on the intensity of infection. Authors should precise the median and/or the range of ct results to give an estimate of the intensity of infection.
2. Did authors compare real time PCR to microscopy results? If yes, results should be shown to support future epidemiological survey based on microscopy as PCR is expensive particularly in large surveys.

Is the work clearly and accurately presented and does it cite the current literature?

Yes

If applicable, is the statistical analysis and its interpretation appropriate?

Yes

Are all the source data underlying the results available to ensure full reproducibility?

Yes

Is the study design appropriate and is the work technically sound?

Partly

Are the conclusions drawn adequately supported by the results?

Yes

Are sufficient details of methods and analysis provided to allow replication by others?

Yes

Reviewer Expertise:

parasitology, molecular biology, epidemiology

We confirm that we have read this submission and believe that we have an appropriate level of expertise to confirm that it is of an acceptable scientific standard, however we have significant reservations, as outlined above.

# Peer review: Reviewer response for version 1

**DOI:** 10.21956/gatesopenres.14306.r29071

**Published:** 2020-07-24

**Authors:** Olusola Ojurongbe

**Affiliations:** 1 Department of Medical Microbiology and Parasitology, Ladoke Akintola University of Technology, Osogbo, Nigeria

This manuscript reported a study carried out in Dosso Region, Niger on Molecular detection of intestinal helminths and protozoa among young children.

 The authors reported high prevalence of *Giardia duodenalis* and generally undernourished  population. The authors concluded that *G. duodenalis* infections may be a significant contributor to child morbidity in the Dosso Region of Niger. However, extremely low sample size of this manuscript makes it difficult to accept this conclusion. These, as well as other major concerns, must be addressed before this manuscript can be considered acceptable for indexing.

 The presentation of the DNA extraction methodology is not clear. The author stated that “the Isolation of total DNA from bulk stool and rectal swabs followed a procedure optimized for the qPCR detection of intestinal helminths”. This procedure was not referenced and and there was no mentioning of kit used. There are many procedure optimized for DNA extraction in stool. The author should properly explain what was done

 The author also should briefly explain what he meant by “multi-parallel” in the result section.

 The authors stated that “Goberi Peulh and Tombo, only contained 1 and 3 total participants” and no infection was observed in theses 2 communities. I will suggest that the names of these communities should not be listed as being free from parasitic infection as the sample size of 1 and 3 is too little to be considered.

 “The prevalence of *G. duodenalis* increased with age, with 88.2% (15/17) testing positive” the author should correct this statement by including age “2 years old” in the sentence.

 I will suggest that the author should not start discussion with “in conclusion”.

 The author reported high prevalence of *G. duodenalis* in rural Niger. While the author attributed low STH and *S. mansoni* to the success of national mass drug administration programs, nothing was discussed as to why *G. duodenalis* infection was high. This should be discussed.

 If according to the author there is a mass administration of PZQ that lead to the absence of STH and S. mansoni, it is not clear why the author is assuming that there will be a higher prevalence of SH in the same community if urine are to be tested. Both SM and SH are susceptible to PZQ. So the explanation of high load or ectopic egg elimination may not be tenable. 

Is the work clearly and accurately presented and does it cite the current literature?

Yes

If applicable, is the statistical analysis and its interpretation appropriate?

Yes

Are all the source data underlying the results available to ensure full reproducibility?

Yes

Is the study design appropriate and is the work technically sound?

Partly

Are the conclusions drawn adequately supported by the results?

No

Are sufficient details of methods and analysis provided to allow replication by others?

No

Reviewer Expertise:

Molecular Parasitology and genetics.

I confirm that I have read this submission and believe that I have an appropriate level of expertise to confirm that it is of an acceptable scientific standard, however I have significant reservations, as outlined above.
